# A12 ROLE OF INTESTINAL EPITHELIAL HNF4A IN PROTECTING AGAINST ACUTE INFLAMMATORY STIMULI

**DOI:** 10.1093/jcag/gwad061.012

**Published:** 2024-02-14

**Authors:** D Pupo Gómez, V Reyes Nicolas, G Marrero Cofino, N Perreault, A Menendez, F Boudreau

**Affiliations:** IBC, Universite de Sherbrooke Faculte de Medecine et des Sciences de la Sante, Sherbrooke, QC, Canada; IBC, Universite de Sherbrooke Faculte de Medecine et des Sciences de la Sante, Sherbrooke, QC, Canada; Immunology and Cell Biology, Universite de Sherbrooke, Sherbrooke, QC, Canada; IBC, Universite de Sherbrooke Faculte de Medecine et des Sciences de la Sante, Sherbrooke, QC, Canada; Immunology and Cell Biology, Universite de Sherbrooke, Sherbrooke, QC, Canada; IBC, Universite de Sherbrooke Faculte de Medecine et des Sciences de la Sante, Sherbrooke, QC, Canada

## Abstract

**Background:**

Inflammatory bowel disease is a group of chronic resulting from complex interactions between host susceptibility genes, the host microbiome, the immune system, and environmental triggers. The gastrointestinal epithelium acts as a barrier against harmful stimuli, and defects in its integrity are a precursor for the development of IBD. Our laboratory has shown that the conditional deletion of *Hnf4a* in the mouse intestinal epithelium leads to chronic intestinal inflammation. However, the functional role of HNF4A on epithelial barrier integrity is still controversial.

**Aims:**

To evaluate the impact of *Hnf4a* deletion on barrier integrity during acute inflammation.

**Methods:**

We used a tamoxifen-inducible Cre^ER^-loxP system to delete *Hnf4a* in the adult intestinal epithelium (*Hnf4a*^*ΔIEC-ind*^). Intestinal permeability was assessed in *Hnf4a*^*ΔIEC-ind*^ and control mice in the collected serum after oral gavage of FITC-dextran. Mice were infected with an invasion-attenuated strain of *Salmonella* Typhimurium (SB103). *S.* Typhimurium loads were scored in the feces, liver, and spleen tissue. Histological analysis was carried out by various staining. Also, we used a model of T-cell-induced enteropathy triggered by a T-cell activating monoclonal anti-CD3ε Ab (145-2C11). Mice were assessed for clinical signs of enteropathy and sacrificed 1-, 3-, and 5 days post-injection. Gene expression of selected targets was studied by RT-qPCR.

**Results:**

FITC-dextran assay showed a significant increase of the intestinal permeability in uninfected mutant mice compared to controls. In contrast, an oral infection with an invasion-deficient *S.* Typhimurium strain did not differentially impact in tissue bacterial loads or epithelial damage between groups. Likewise, the loss of epithelial HNF4A led to increased Muc2 transcripts expression in the ileum of infected mutant mice but not in the colon. Histological examinations at four days post-infection showed an elevated number and size of goblet cells in the ileal crypts of mutant mice. Bacterial localization using the general bacterial probe EUB388-cy3 (red) staining showed that infected mutant mice tended to enhance barrier mucus layer thickness compared to the controls. On the contrary, acute T cell activation with anti-CD3 mAb induced a significant increase of mRNA expression of Cxcl1 in the small intestine of mutant mice. H&E staining showed elongated ileal crypts in mutant mice compared to controls after anti-CD3 injection.

**Conclusions:**

Altogether, these results support that HNF4A could play a crucial adaptive role in modulating the small intestine epithelial barrier function and controlling the immune system during acute inflammatory stress.

**Funding Agencies:**

CCC, CIHR

